# Reversible GABAergic dysfunction involved in hippocampal hyperactivity predicts early-stage Alzheimer disease in a mouse model

**DOI:** 10.1186/s13195-021-00859-8

**Published:** 2021-06-14

**Authors:** Yang Li, Ke Zhu, Ning Li, Xiaotong Wang, Xuansheng Xiao, Linying Li, Lijuan Li, Ying He, Jinglan Zhang, Jiaoyang Wo, Yanqiu Cui, Haixia Huang, Jianliang Zhang, Wei Wang, Xiaomin Wang, Yan Zheng

**Affiliations:** 1 Key Laboratory for Neurodegenerative Disorders of the Ministry of Education, Capital Medical University, Beijing, 100069 China; 2 Beijing Institute for Brain Disorders, Capital Medical University, Beijing, 100069 China; 3 Department of Physiology and Pathophysiology, School of Basic Medical Sciences, Beijing, China; 4 Beijing Key Laboratory of Neural Regeneration and Repair, Beijing, China

**Keywords:** Neuronal hyperactivity, Synaptic transmission, Alzheimer’s disease, GABA_A_ receptors, 5XFAD mouse

## Abstract

**Background:**

Neuronal hyperactivity related to β-amyloid (Aβ) is considered an early warning sign of Alzheimer disease (AD). Although increasing evidence supports this opinion, the underlying mechanisms are still unknown.

**Methods:**

Here, we recorded whole-cell synaptic currents and membrane potentials using patch clamping of acute hippocampal slices from human amyloid precursor protein (APP)/presenilin-1 transgenic (5XFAD) mice and their wild-type littermates. Biochemical methods, electron microscopic imaging, behavioral tests, and intraventricular drug delivery applied with osmotic pumps were used in this study.

**Results:**

We confirmed hyperactivity of hippocampal CA1 pyramidal neurons in 5XFAD mice using whole-cell electrophysiological recording at 2.5 months old, when local Aβ-positive plaques had not developed and only mild cognitive dysfunction occurred. We further discovered attenuated inhibitory postsynaptic currents and unchanged excitatory postsynaptic currents in CA1 pyramidal neurons, in which the intrinsic excitability was unchanged. Moreover, the density of both γ-aminobutyric acid A (GABA_A_) receptor subunits, α1 and γ2, was reduced in synapses of the hippocampus in transgenic mice. Intriguingly, early intervention with the GABA_A_ receptor agonist gaboxadol reversed the hippocampal hyperactivity and modestly ameliorated cognitive performance in 5XFAD mice under our experimental conditions.

**Conclusions:**

Inhibitory postsynaptic disruption critically contributes to abnormalities in the hippocampal network and cognition in 5XFAD mice and possibly in AD. Therefore, strengthening the GABAergic system could be a promising therapy for AD in the early stages.

**Supplementary Information:**

The online version contains supplementary material available at 10.1186/s13195-021-00859-8.

## Background

Alzheimer disease (AD) is a common cause of dementia that features extracellular β-amyloid (Aβ) deposition and intracellular neurofibrillary tangles [1] and has a pathogenesis that is not clearly understood. Although overproduction or accumulation of Aβ, which is generated from its precursor protein (APP), is considered to have a leading role in AD pathophysiology [2], most clinical trials targeting this mechanism have failed, indicating that the mechanisms in the early stages of AD need further exploration.

The optimal function of a neural circuit is dependent on the effective information processing of individual synapses. The ensemble of excitatory and inhibitory inputs to postsynaptic action potential (AP) conversion determines specialized circuit functions during cognitive processes. The hippocampus is responsible for computing contextual and spatiotemporal information and is an indispensable brain region for learning and memory processes. An imbalance of the inhibitory and excitatory synaptic inputs in the hippocampus results in disrupted AP outputs, mainly from pyramidal neurons [3–5], which play a key role in computing various signals in learning behaviors. Derangement of the neural circuitry and subsequent epileptiform activity in the hippocampus is considered a critical event involved in AD [6–9].

Although functional synaptic failure and related neural circuit aberrance in some regions of the brain in AD are widely accepted as critical events in the whole process of AD [10–14], the exact mechanism underlying this network aberrance has yet to be elucidated. It has been assumed that hippocampal neuronal hyperactivity associated with epileptic seizures is a very early functional impairment, is even a surrogate marker in patients with AD and animal models, and occurs before extracellular Aβ deposition [9, 15–18]. Yet, how presynaptic or postsynaptic components contribute to neuronal hyperactivity and ultimate neural network disintegration and cognitive disorder in AD is poorly understood.

Our previous observations and other studies have revealed attenuated excitatory synaptic transmission [19], a decline in long-term potentiation [20], and abnormal neuronal activity [21, 22] in the brains of the 5XFAD mouse model carrying human mutations of APP/presenilin-1 (PS1). However, the early phenotypes related to AD pathology and the mechanism involved in brain region-specific neuronal hyperactivity in AD remain to be clarified. In the present study, we sought to explore the mechanism involved in hippocampal neuronal hyperactivity that is correlated with cognitive deficits in early disease stages in an AD mouse model. First, we confirmed that hyperactivity of pyramidal neurons in CA1 preceded Aβ-related pathology and was accompanied by mild cognitive impairments in 5XFAD transgenic mice. Further investigation revealed that the aberrant AP output from CA1 pyramidal neurons was driven by γ-aminobutyric acid A (GABA_A_) receptor-mediated inhibitory synaptic decline. Most importantly, GABA_A_ receptor agonist could reverse the decline in cognitive performance in the mouse model. Thus, we propose that functional decline in inhibitory synaptic transmission critically contributes to hippocampus hyperactivity and cognitive deficits in the early stages of AD-like conditions. Therefore, activating GABA_A_ receptors could be a promising path to early intervention of AD.

## Methods

### Animal model and brain slices recordings

APP/PS1 (5XFAD) double transgenic mice (006554, Jackson Laboratory) carrying human APP and PS1 transgenes containing five FAD mutations (APPSwFlLon, PSEN1*M146L*L286V) under the transcriptional control of the neuron-specific mouse Thy-1 promoter were bred in strict accordance with Chinese regulations involving animal protection. The animal experiment was approved by the animal ethics committee of Capital Medical University. Mice were maintained by crossing heterozygous transgenic mice with C57BL/6 wild-type breeders. We used female heterozygous mice in electrophysiological recordings and male heterozygous in behavioral tests, aged between 2.5 and 3.5 months, and non-transgenic wild-type age-matched littermates served as the control group.

Under chloral hydrate (I.P. 300 mg/kg) anesthesia, mice were transcardially perfused with cutting solution, and the brains were immediately removed and cut with a vibratome (Leica, VT1200S). For cell recordings, the slices were placed in a recording chamber constantly perfused with ACSF (Supplementary Information).

### Subcellular fractionation

Shared with the above electrophysiological experiment when needed. Fractions were prepared as described previously with a few modifications (Supplementary Information).

### Western blotting

Equal amounts of proteins, which were quantified with BCA protein assay kit (23225, Pierce TM) from WT and FAD fractions were subjected to 10% SDS–polyacrylamide gels and transferred to nitrocellulose membranes. Then, the membranes were incubated with various primary antibodies (Supplementary Information).

### ELISA

The mouse brain tissues were homogenized in cold RIPA buffer. The supernatant and the pellet were separated for soluble and insoluble Aβ detections, respectively, with kits (KHB3441, KHB3481, Invitrogen) (Supplementary Information).

### Biocytin labeling and immunostaining

Neuronal morphology and spine density of CA1 neurons were determined by adding biocytin (0.04%) in the pipette solution during whole-cell recording. Specific antibodies recognizing subunits of GABA_A_ receptors or AMPA receptors were used in immunofluorescent detection. Avidin–biotin complex (ABC) staining was used to determine Aβ-positive signals (Supplementary Information).

### Electron microscopic imaging

Brain tissues were punched and subjected to sequential preparation for imaging of transmission electron microscope (HITACHI, JAPAN, HT7700) (Supplementary Information).

### Behavior and drug treatment

Paradigms of contextual fear conditioning (CFC) and episodic-like memory were utilized to estimate hippocampus-dependent cognition of mice. For drug treatment in vivo, intraventricular delivery was performed to mimic local perfusion as applied in slice recording (Supplementary Information).

### Statistical analysis

All data were first tested for normality. Datasets that passed the normality test were subjected to parametric tests with unpaired t-test for two groups comparison or one/two-way ANOVA for more than two groups comparison. The data which did not pass the normality test were analyzed by non-parametric tests. The analysis software was GraphPad Prism 8.0.1 (detailed statistical reports can be found in Supplementary Table 1).

## Results

### CA1 neuronal hyperactivity preceded local Aβ deposition and was accompanied by mild cognitive decline in 5XFAD mice

**Fig. 1:**
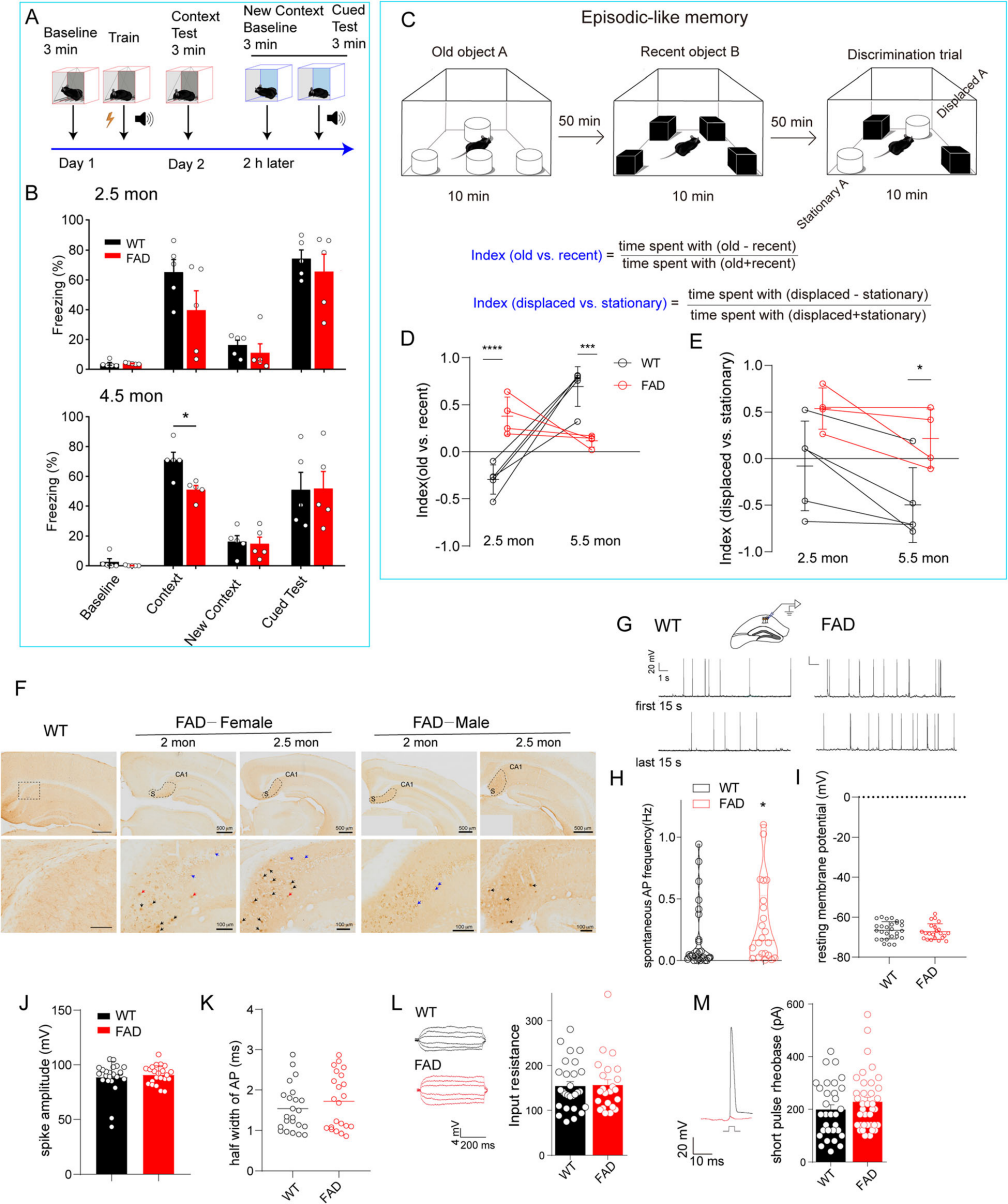
Hippocampal CA1 pyramidal neurons exhibited hyperactivity accompanied with few extracellular Aβ plaques in 5XFAD mice with mild cognitive decline. **A** Schematic diagram of contextual fear conditioning (CFC). **B** 5XFAD mice showed a significant reduction in freezing time at 4.5 months old (51.08 ± 2.76% vs. 71.14 ± 5.052%) while remained comparable level with WT mice at 2.5 months old (39.62 ± 29.4% vs. 65.19 ± 18.88%) in context test. Data are analyzed by two-way ANOVA with Bonferroni’s multiple comparisons test, mean ± SEM, *p < 0.05 vs. WT, n = 5 mice per group. **C** novel object recognition paradigm reflecting episodic-like memory. **D**, **E** The index (old vs. recent) indicating the ability to identify objects (**D**) and the index (displaced vs. stationary) indicating episodic memory of mouse (**E**) were subjected to statistical analysis. *p < 0.05, ***p < 0.001, ****p < 0.0001 vs. WT with two-way ANOVA, followed by Sidak’s multiple comparisons test, n = 5 in WT, n = 4 in FAD. Interaction (age × genotype) is significant (***p = 0.0002) in **D** and not significant in **E**. **F** Intraneuronal Aβ (stained with 6E10 antibody) signals were found in the cortex and hippocampus. The subiculum areas were surrounded by dashed lines (above), bar scale 500 μm. Obvious Aβ deposition was shown in a locally enlarged image of the subiculum (bottom) in a 2–2.5-month-old 5XFAD mouse. Red arrow: primary plaques; black arrow: canonical plaques; blue arrow: intraneuronal Aβ deposition. Bar scale 100 μm. **G** Action potential of the CA1 pyramidal neuron in WT (left) or 5XFAD mice (right) (female at 2.5 months old) was recorded with whole-cell current-clamp recording. The traces show the first 15 s and the last 15 s in at least 5 min recording time. **H**–**K** The spontaneous action potential (sAP) events during recording time (**H**), the resting membrane potential (**I**), the amplitude of spike (**J**), and the half-width of AP were detected automatically with Clampfit10.4. Unpaired Student’s t-test was used to compare these two groups, and the Mann–Whitney test was used in **H**, *p = 0.0380, n = 27 neurons/13 mice for WT; n = 22 neurons/10 mice for FAD, effect size is 0.299. In **J** and **K**, unpaired t-test with Welch’s correction was applied. Except for **H**, all values are presented as mean ± SEM. **L** Subthreshold stimulation was constituted of current injections from − 30 to + 30 pA in a duration of 500 ms, and the input resistance of CA1 pyramidal neurons in WT and 5XFAD mice was determined by Ohm’s law. **M** Step current injections (increment 10 pA) crossing the threshold were performed to determine the short-pulse rheobase of neurons. The red trace indicates the last subthreshold voltage response, and the black trace depicts the first firing triggered by suprathreshold current injection. The dots indicate individual neurons recorded, n = 28 (FAD), n = 23 (WT) in **L**; n = 31 (WT), n = 37 (FAD) in **M**. Unpaired Student’s t-test was used in **L** and **M**

We first examined the phenotypical characteristics of WT and 5XFAD mice. The CFC was applied to differentiate hippocampus-dependent contextual fear memory, showing significant attenuated performance in 5XFAD mice at the age of 4.5 months compared with their WT littermates. At 2.5 months old, although the 5XFAD mice had no significant change in freezing percentage on average, these mice varied individually (Fig. 1A, B), indicating differing stages of disease progression. Interestingly, in episodic-like memory tests (Fig. 1C), although at 5.5 months old the transgenic mice showed considerable impairment in discrimination between familiar and novel objects (Fig. 1D), at 2.5 months old, they spent more time in contact with familiar objects than with novel ones, in contrast to WT controls (Fig. 1D). This result may reflect mild symptoms of AD in which recent memory reflects early memory impairment in the AD process [23]. Furthermore, we used an index of displaced familiar objects vs. stationary familiar objects to evaluate cognition with contextual information. The 5XFAD mice at 5.5 months old showed an impaired ability to discriminate stationary object A, which carried “old” information in association with episodic-like memory (Fig. 1E, 5.5 mon). Intriguingly, despite showing non-significance compared with WT control mice, all transgenic mice at 2.5 months old had no ability to recognize the stationary object A (Fig. 1E, 2.5 mon). These results imply that hippocampal functions would have been damaged at 2.5 months of age in the 5XFAD mice. In addition, the 5XFAD mice at 2.5–3.5 months old exhibited a dramatic increase in insoluble Aβ (Supplementary Figure 1), but we barely observed accumulation of Aβ in the hippocampal CA1 region, despite intracellular and extracellular deposition in the subiculum (Fig. 1F), which is an output region of CA1.

At ages 2.5–3 months, male 5XFAD mice showed a pathological phenotype that was comparable with that of female 5XFAD mice. We therefore performed physiological experiments using female mice and tested behaviors using male mice to avoid possible effects of the menstrual cycle in the present study. To evaluate the firing state of CA1 pyramidal neurons in the mice, we prepared acute hippocampal slices and recorded the spontaneous action potential (sAP) with a whole-cell current clamp. The frequency of sAP in the CA1 pyramidal neurons of 5XFAD mice at 2.5 months old was moderately enhanced compared with WT mice (p = 0.038; Fig. 1G, H) while becoming more pronounced with advancing age (Supplementary Figure 2). Considering that the resting membrane potential (Fig. 1I), amplitude of the action potential (AP) spike (Fig. 1J), and half-width of the AP (Fig. 1K) were unchanged, these two genotypic mice have the same basic physiologic firing properties. In a neuron, a higher input resistance may result in the depolarization threshold in the AP being easily reached. Therefore, we measured input resistance by applying subthreshold step-current injections with a duration of 500 ms, and rheobase with brief step-current injections with a duration of 3 ms sequentially. Consistently, both the input resistance and the rheobase of CA1 pyramidal neurons in 5XFAD mice were comparable with that of WT mice (Fig. 1L, M). These results confirmed that neuronal hyperactivity in the hippocampal CA1 precedes local extracellular Aβ accumulation in the AD mouse model. In addition, the intrinsic neuronal excitability was unchanged by the APP/PS1 transgenic background at this age, suggesting synaptic dysfunction underlying CA1 neuronal hyperactivity in the early stage of AD pathophysiology.

### Activity-dependent excitatory synaptic transmission to CA1 pyramidal neurons of 5XFAD mice was unchanged

**Fig. 2:**
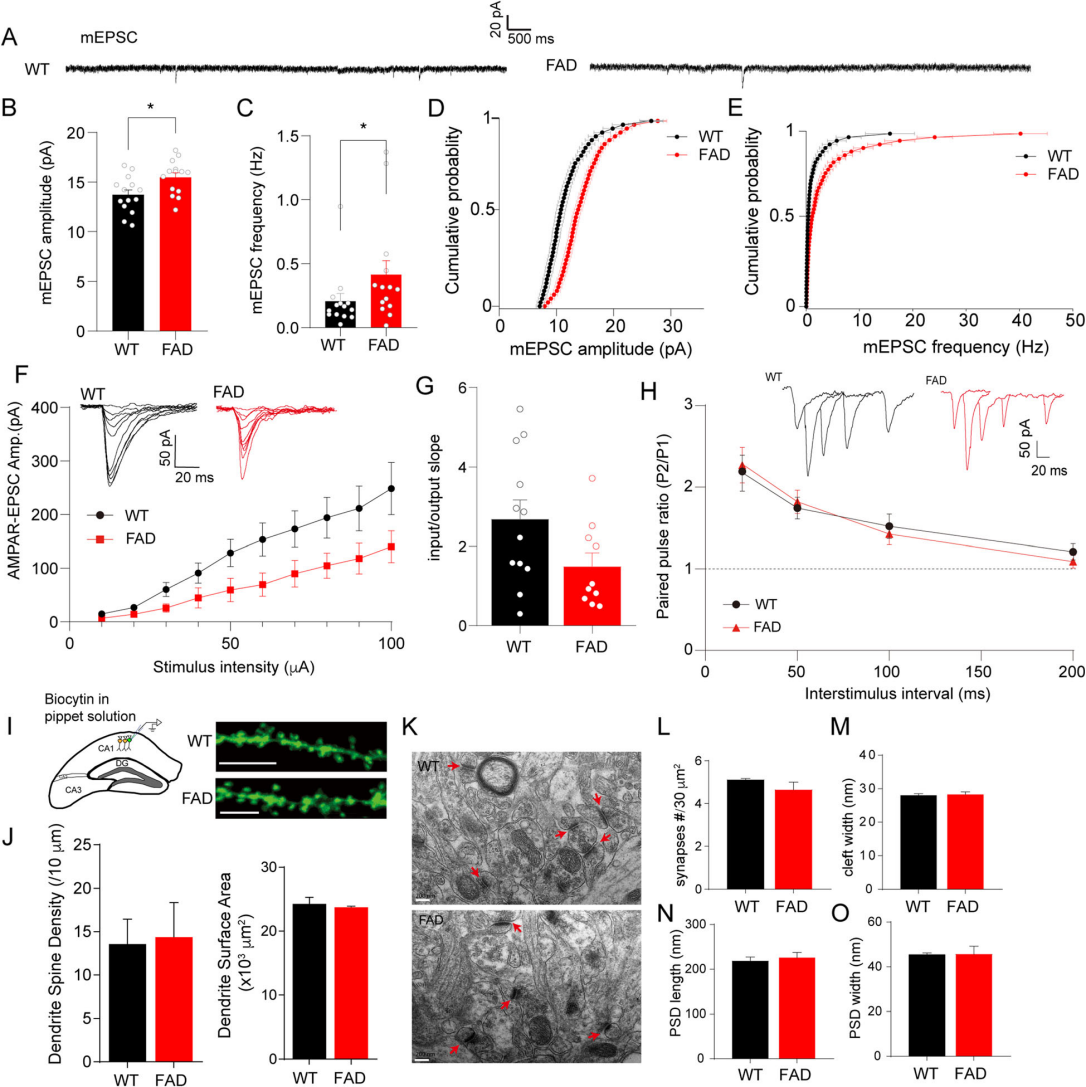
The activity-dependent excitatory synaptic responses and spine morphology exhibited no obvious change in CA1 pyramidal neurons of 5XFAD mice at 2.5 months old. **A** Representative traces show miniature EPSCs (mEPSCs) in CA1 pyramidal neurons from WT or 5XFAD mice. **B**, **C** Amplitude (**B**) and frequency (**C**) of mEPSCs were analyzed, n_(WT)_ = 14 neurons/7 mice, n_(FAD)_ = 14 neurons/9 mice. For amplitude, the data were subjected to a two-tailed unpaired t-test, *p = 0.0153 for FAD (15.47 ± 0.46 pA) vs. WT (13.72 ± 0.49 pA); for frequency, the data did not pass normality tests and were subjected to a Mann–Whitney test, *p = 0.0301 for FAD vs. WT. **D**, **E** Cumulative probability plots show the distribution of amplitude and frequency of mEPSCs in WT and 5XFAD neurons. **F** Analysis of input–output (I-O) relationship between AMPAR EPSCs and incremental stimulation intensities (in μA) 10, 20, 30, 40, 50, 60, 70, 80, 90, and 100 (WT, 14.67 ± 3.90, 26.98 ± 5.44, 60.64 ± 13.2, 91.02 ± 18.94, 128.40 ± 25.29, 153.60 ± 30.76, 173.30 ± 33.78, 194.10 ± 37.97, 211.50 ± 41.79, 248.66 ± 48.79, respectively, n = 12 neurons/7 mice; FAD, 6.88 ± 1.07, 14.01 ± 3.66, 25.81 ± 7.81, 44.86 ± 18.73, 59.63 ± 21.64, 89.94 ± 24.67, 104.79 ± 23.54, 118.20 ± 28.88, 140.25 ± 29.61 pA, respectively, n = 10 neurons/6 mice) with two-way ANOVA analysis followed by Bonferroni’s multiple comparisons test, and insets show representative traces of WT and FAD slices. **G** Input/output slope of each cell was calculated and analyzed with unpaired Student’s t-test, *p* = 0.066. **H** Representative traces (inset) and plot show PPRs at interstimulus intervals of 20, 50, 100, and 200 ms with unpaired Student’s t-test (WT, 2.18 ± 0.23, 1.74 ± 0.13, 1.52 ± 0.15, 1.21 ± 0.10, respectively, n = 17 neurons; FAD, 2.85 ± 0.42, 1.82 ± 0.14, 1.43 ± 0.13, 1.09 ± 0.08, respectively, n = 10 neurons, *p* = 0.14, 0.71, 0.68, 0.39 for each interstimulus interval). **I** Biocytin was added in pipette solution and injected intracellularly during whole-cell recording (left diagram), followed by staining with streptavidin-coupled Alexa 488. Right representative images show the sections of dendrites from WT and FAD neurons, bar scale 5 μm. **J** Biocytin-positive neurons were reconstructed with the Imaris software, and dendrite spine density and dendrite surface area were calculated and subjected to analysis, n = 3 mice for WT or FAD genotype. **K** Punched brain tissues containing CA1 area were subjected to electron microscopic observation. Red arrows show synapses, and the bar scale is 200 nm. **L**–**O** The number of synapses per 30 μm^2^ (**L**), synaptic cleft width (**M**), length (**N**), and width (**O**) of postsynaptic density (PSD) were calculated by the ImageJ software (20 synapses/3 mice in WT; 18 synapses/3 mice in FAD) and put into unpaired Student’s t-test. In **B**, **C**, and **G**, the dots show individual cells; in **J**, **L**–**O**, the dots show individual mice. All values are presented as mean ± SEM

As the intrinsic properties of CA1 pyramidal neurons were not altered, we determined whether inhibitory or excitatory synaptic transmission to CA1 pyramidal neurons contributed to the hyperactivity in the transgenic hippocampus. We first measured mEPSCs in CA1 pyramidal whole-cell recordings. The slight increases in amplitude and frequency of mEPSCs of CA1 pyramidal neurons in 5XFAD slices (Fig. 2A–C), together with the cumulative distribution of these currents that had shifted to higher amplitude or frequency of mEPSC events compared with that of WT slices (Fig. 2D, E), indicated little enhanced spontaneous excitatory synaptic transmission. As the miniature synaptic events were recorded in the presence of TTX, which blocked activity-dependent synaptic transmission, we further recorded evoked AMPA receptor-mediated EPSCs (AMPAR-EPSCs) at the holding potential of − 60 mV, responding to incremental stimulus intensities applied at Schaffer collateral. In contrast to the changes in miniature events, as stimulus intensity increased, CA1 pyramidal neurons in the 5XFAD brain slices showed a decreasing trend, but with no statistical change in AMPAR EPSC amplitude (Fig. 2F, G). The paired-pulse ratios (PPRs) at multiple interstimulus intervals (Fig. 2H), that is, inversely correlated with presynaptic transmitter release probability [24], were also not affected. The result of the activity-dependent synaptic responses suggested that the basic excitatory synaptic transmission to CA1 pyramidal neurons can be assumed to be compensatory in early stages of the AD-like process. To determine whether the dendritic morphology of CA1 pyramidal neurons was changed, we included biocytin in the intracellular recording pipette during whole-cell recording and performed observations with confocal microscopy (Fig. 2I). The 5XFAD neurons showed dendrite spine density and surface area comparable to those of WT neurons (Fig. 2J), supporting our hypothesis that the enhancement of miniature excitatory synaptic events was a secondary and functional response of CA1 neurons in the AD context. Observations by electron microscopy revealed supportive evidence that the number of asymmetric synapses, the cleft width of synapses, and the length and width of postsynaptic density were all intact in the transgenic mouse hippocampus (Fig. 2K–O).

### Inhibitory synaptic transmission to CA1 pyramidal neurons was attenuated in the brain of 5XFAD mice

**Fig. 3:**
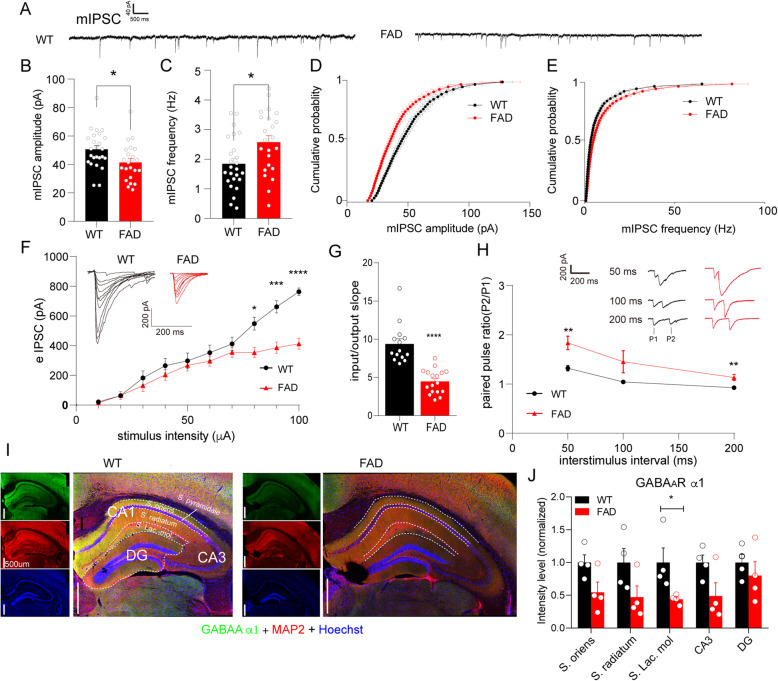
The inhibitory synaptic response of the CA1 pyramidal neuron was attenuated in the 5XFAD mouse model. **A** Representative traces showing miniature IPSCs (mIPSCs) in the CA1 pyramidal neuron of WT and 5XFAD mice. **B**, **C** Amplitudes (**B**) and frequency (**C**) of mIPSCs, n_(WT)_ = 25 neurons/11 mice, n_(FAD)_ = 23 neurons/8 mice, were subjected to unpaired Student’s t-test, *p = 0.0192 for amplitude, *p = 0.014 for frequency. **D**, **E** Cumulative distribution plots for amplitude (**D**) and frequency (**E**) of mIPSCs in WT and 5XFAD mice. **F** Input–output (I-O) relationship between evoked IPSCs (eIPSCs) and incremental stimulation intensities (in μA) 10, 20, 30, 40, 50, 60, 70, 80, 90, and 100 (WT, 21.52 ± 3.25, 63.56 ± 13.96, 182.98 ± 47.65, 265.61 ± 49.33, 298.14 ± 52.68, 352.74 ± 50.99, 412.40 ± 45.03, 548.96 ± 43.79, 662.15 ± 41.27, 764.10 ± 25.28 pA, respectively, n = 14 neurons/4 mice; FAD, 14.50 ± 4.44, 64.64 ± 26.56, 131.58 ± 39.08, 204.07 ± 37.37, 267.47 ± 37.74, 296.47 ± 34.27, 355.09 ± 34.99, 353.79 ± 34.24, 386.77 ± 37.57, 412.59 ± 37.39 pA, respectively, n = 17 neurons/7 mice; with two-way ANOVA analysis followed by Bonferroni’s multiple comparisons test, *p < 0.05 under 80 μA stimulus intensity, ***p < 0.001 under 90 μA stimulus intensity, ****p < 0.0001 under 100 μA stimulus intensity), and the insets show representative traces of WT and FAD slices. **G** Input/output slope of each cell was analyzed with unpaired Student’s t-test, ****p < 0.0001. **H** Representative traces (insets) and plot showing the paired-pulse ratio (P2/P1, PPR) at interstimulus intervals of 50, 100, and 200 ms (WT, 1.32 ± 0.05, 1.05 ± 0.03, 0.93 ± 0.03, respectively, n = 15 neurons/4 mice; FAD, 2.32 ± 0.26, 1.84 ± 0.14, 1.45 ± 0.23, 1.14 ± 0.06, respectively, n = 16 neurons/6 mice, with unpaired Student’s t-test, and Welch’s correction was applied in 50 ms interval, *p* = 0.0008, 0.11, 0.0044, respectively). **I** GABA_A_ receptor subunit α1 was immunostained (green) in WT and 5XFAD brain sections, and regions of interest (ROI) in the hippocampus were separated by a dashed line. MAP2 immunostaining (red) was used as internal control, and the cell nucleus was labeled by Hoechst (blue). The bar scale is 500 μm. **J** Mean gray value of α1-positive intensity (divided by ROI area) measured with the ImageJ software was subjected to unpaired Student’s t-test for each subregion of the hippocampus, *p < 0.05 vs. WT, n = 4 mice per group

In contrast to mEPSCs, the mIPSCs of FAD CA1 pyramidal neurons displayed a decrease in amplitude and a slight increase in frequency as compared with WT neurons (Fig. 3A–C). The cumulative distribution of mIPSCs in 5XFAD neurons consistently displayed a shift to lower amplitude or higher frequency (Fig. 3D, E). This result suggested that inhibitory synaptic transmission is probably damaged in early stages of the AD-like process. To further determine activity-dependent inhibitory synaptic transmission, we recorded evoked GABA_A_ receptor-mediated IPSC (eIPSC) current using electrostimulation at Schaffer collateral in the absence of TTX, with QX314 in a pipette to block the voltage-gated sodium channel in the recorded cell. As expected, we observed a suppressed response to higher stimulus intensities (80–100 μA) and a pronounced reduction in the input/output slope of eIPSCs in 5XFAD slice neurons compared with WT neurons (Fig. 3F, G). Given that the response of the postsynaptic membrane can be attributed to either a presynaptic release probability or function of postsynaptic receptors, we checked the PPRs of eIPSCs in these neurons. Notably, the 5XFAD neurons had higher PPRs than those of WT neurons (Fig. 3H), indicating decreased activity-dependent presynaptic GABA release onto 5XFAD CA1 pyramidal neurons. As GABA_A_ receptors mediate IPSCs, we also conducted immunofluorescent imaging of GABA_A_ receptor subunit α1. Intriguingly, α1 expression displayed a regional selective decrease in the stratum lacunosum-moleculare (SLM) of CA1, with a normal level of MAP2 expression in the 5XFAD brain (Fig. 3I, J). Given that the SLM is a subfield, the apical dendrites of the CA1 pyramidal neurons are located in and accept direct inputs from the entorhinal cortex [25], which is one of the earliest involved regions of the brain in AD [1]. Our result implies that the integration of inhibitory synaptic transmission in CA1 would most likely be damaged by the early AD-related microenvironment.

GABA_A_ receptors can mediate either phasic synaptic transmission or tonic inhibition via activation of extrasynaptic GABA_A_ receptors caused by GABA release from reactive astrocytes owing to AD pathology [26, 27]. Therefore, we recorded tonic current induced by the GABA_A_ blocker bicuculline in the presence of GABA and GABA uptake inhibitor. We found that the AD transgenic background neither increased the degree of the tonic response of CA1 pyramidal neuron nor affected the astrocytic immunoreactivity (Supplementary Figure 3).

### GABA_A_ receptor sensitivity to agonist was reduced, accompanied by decreased subunit density in the synapses in the hippocampus of 5XFAD mice

**Fig. 4:**
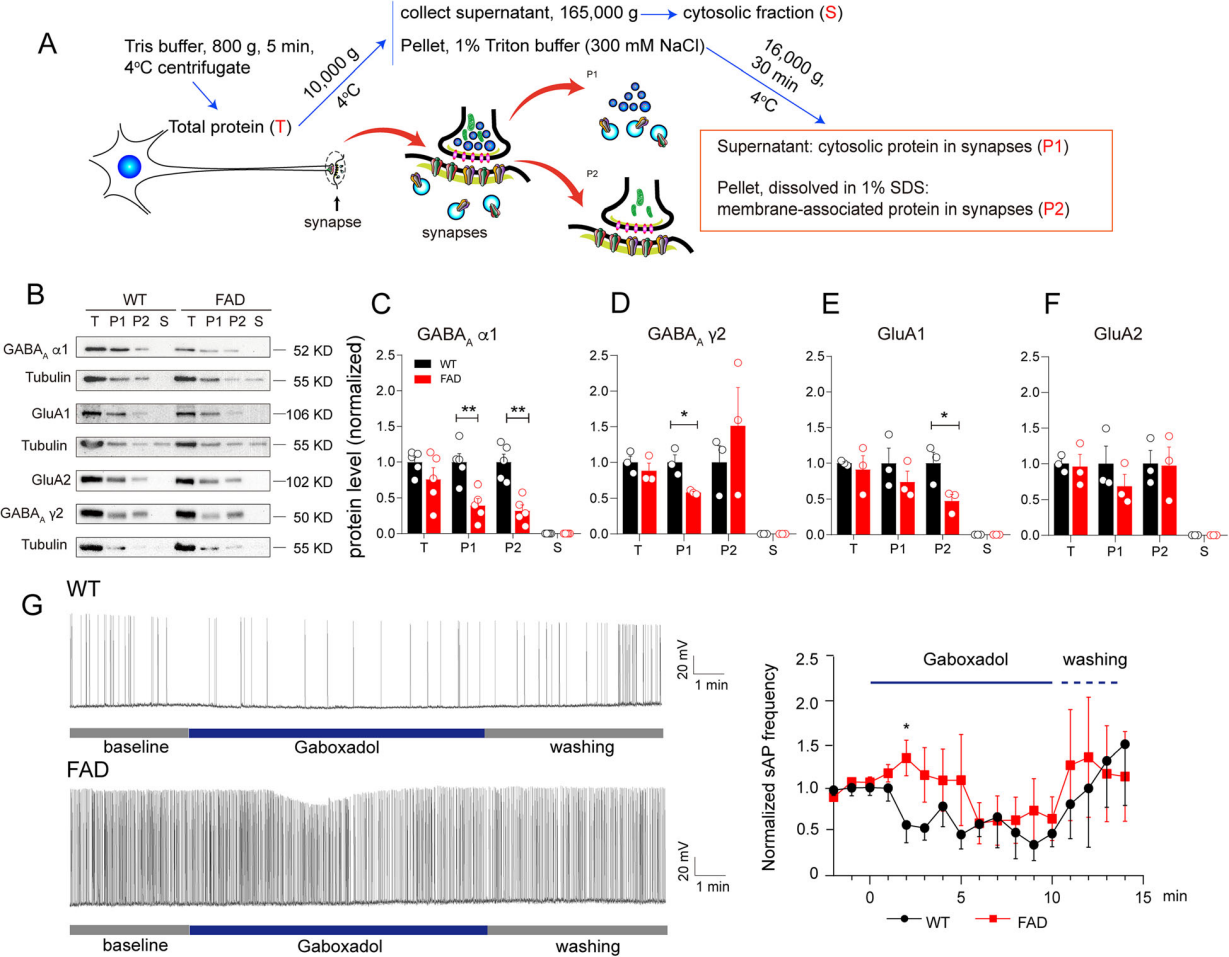
The attenuation of GABA_A_ receptor sensitivity to agonist was accompanied with declined GABA_A_ subunits localization in the synapses of the 5XFAD hippocampus. **A** Diagram for preparation of synaptic fractions. **B** Representative bands of western blotting for fractions from the WT and 5XFAD mouse hippocampus. C–F Normalized protein levels of GABA_A_ α1 subunit (**C**), GABAA γ2 subunit (**D**), GluA1 (**E**), and GluA2 (**F**) distributed in each fraction (T, P1, P2, S, respectively) were subjected to unpaired Student’s t-test, *p < 0.05, **p < 0.01 vs. WT. The dots show the number of mice in each group, n = 5 mice/group in C, n = 3 mice/group in **D**–**F**. All values are presented as mean ± SEM. **G** GABA_A_ receptor agonist, gaboxadol (GBX), in the final concentration of 5 μM was added in extracellular ACSF solution during slice whole-cell recording. The spontaneous action potential (sAP) of CA1 pyramidal neurons in WT or 5XFAD mouse slices was recorded, and the frequency was normalized to baseline. Values were analyzed by unpaired Student’s t-test for each time point, n_(WT)_ = 5, n_(FAD)_ = 8, *p = 0.0289 for 2 min

We next asked whether the decreased inhibitory synaptic transmission was due to a decrease in the total level of these receptors or only their altered distribution in the synapses. To answer this question, we separated the synapses from total hippocampal extracts of 2.5-month-old 5XFAD and WT mice. The distribution of GABA_A_R α1 in both cytosolic fractions (P1) and membrane-associated fractions (P2) in 5XFAD hippocampal synapses was significantly reduced, with normal total protein levels, as compared with WT mice (Fig. 4A–C). Because α1/β/γ2 containing heteropentameric GABA_A_ receptors are predominantly located in brain synapses [28, 29], we also examined the γ2 subunit expression, which showed an obvious reduction in cytosolic fractions but had normal levels of membrane fractions compared with WT mice (Fig. 4D). In the same experimental system, AMPARs subunits, GluA1 and GluA2, exhibited normal levels of cytosolic and membrane fractions, except for a lower membrane fraction level in GluA1 than that of WT mice (Fig. 4E, F). However, we found no obvious change in GluA1 immunostaining in the hippocampus of 5XFAD mice (Supplementary Figure 4). These findings indicated that the GABA_A_ receptors displayed a decline in synaptic distribution, with a normal reserve pool, and probably maintained repairable synaptic function. Therefore, we added a GABA_A_ receptor agonist, GBX in ACSF, to treat current-clamped neurons and observed a retarded response of 5XFAD neurons in discharging change (after 4 min of perfusion with ACSF-containing GBX); WT neuron displayed a fast drop in firing frequency after 2 min of GBX treatment (Fig. 4G). Despite the delayed response to GBX, 5XFAD abnormal firing was eventually repressed by the GABA_A_ agonist, suggesting on the one hand that the functional decline in GABA_A_ receptors may play a key role in CA1 neuronal hyperactivity and, on the other hand, that AD-like phenotypes in the early stages could be intervened using GABA_A_ receptor agonists.

### Activating GABA_A_ receptors modestly ameliorated cognitive performance in 5XFAD mice

**Fig. 5:**
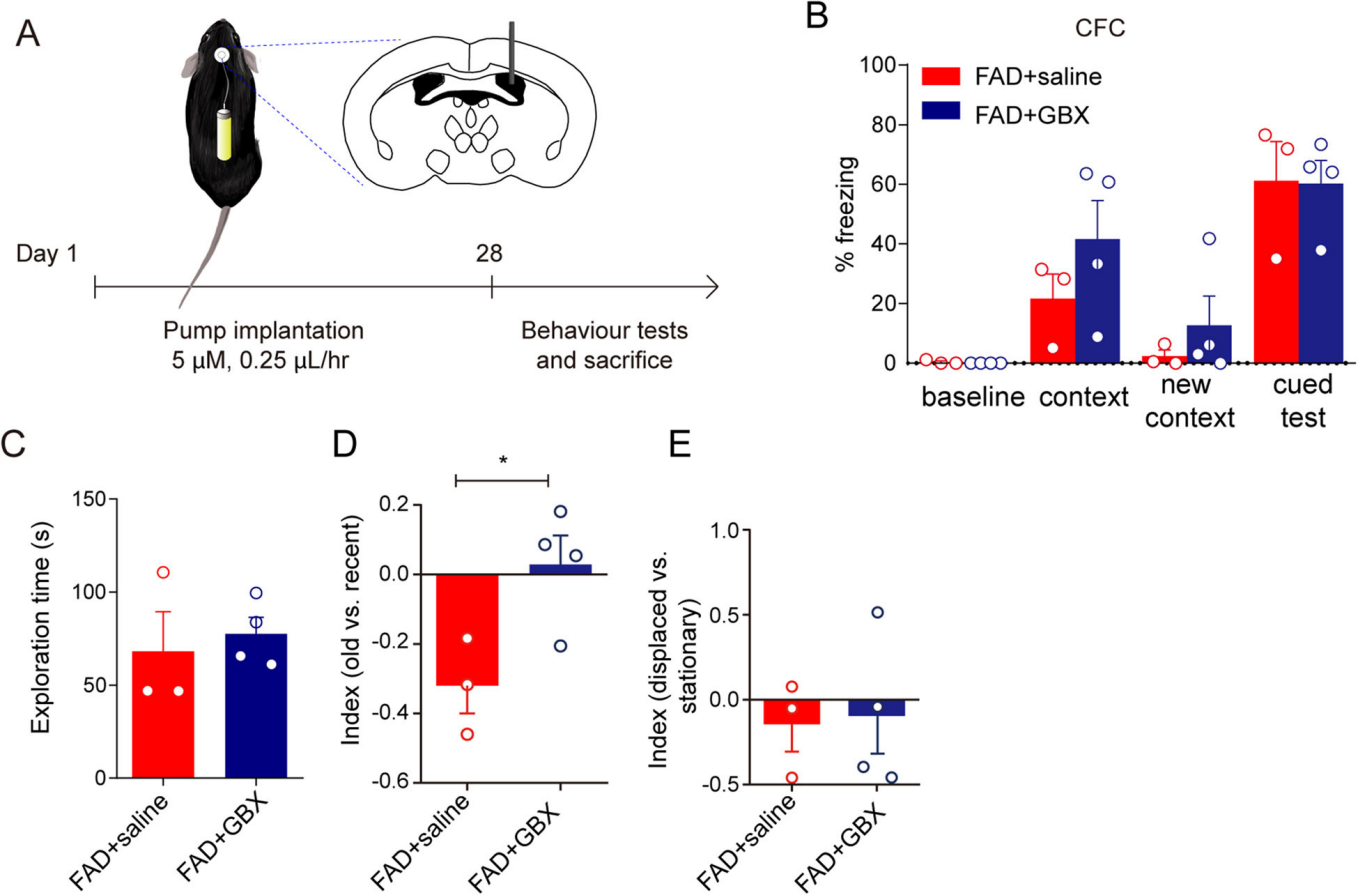
GABA_A_ agonist, GBX, treatment modestly ameliorated cognitive performance of 5XFAD mouse model. **A** The schematic diagram for the timeline of drug treatment and behavioral tests. The 5XFAD male mice at 3.5 months old were randomly divided into two groups for unilateral intraventricular drug delivery. The GBX (5 μM) or saline was loaded into an osmotic pump, which provided drug/saline at a speed of 0.25 μL/h to right lateral ventricle. The osmotic administration lasted for 28 days. **B** Contextual fear conditioning (CFC) test was performed to determine fear memory ability in GBX-treated mice and saline-treated mice. Two-way ANOVA followed with Bonferroni’s multiple comparisons test was used, mean ± SEM, n = 3 in saline-treated mice, n = 4 in GBX-treated mice. **C**–**E** In episodic-like memory test, the exploration time (**C**, the total time taken in discrimination trial), old vs. recent index (**D**, time spent with (old − recent)/time spent with (old + recent)), displaced vs. stationary index (**E**, time spent with (displaced − stationary)/time spent with (displaced + stationary)). All values are presented as the mean ± SEM and analyzed by unpaired Student’s t-test, *p = 0.0321 in **D**, and the dots show the number of mice used in each group, n = 3 in saline-treated mice, n = 4 in GBX-treated mice

We further asked if the functional decline in inhibitory synaptic transmission was specifically a critical causative event in AD progression and whether activating the GABA_A_ receptors could rescue cognitive behaviors in the AD model in the early stages. Thus, we treated 5XFAD mice at 3.5 months old with GBX, which is a metabolically stable GABA_A_ receptor agonist that has been used in the treatment of sleep order [30], or with saline to evaluate cognitive performance in CFC and episodic-like memory tasks. We used osmotic pumps to deliver the drug in local concentrations of 5 μM, which is equal to that used in slice perfusion, at a speed of 0.25 μL/h to provide continuous unilateral intraventricular drug treatment; the treatment lasted for 28 days (Fig. 5A). Under these conditions, the hippocampus should be the main area receiving the drug effect. In the CFC test, there was no significant difference in the percent of freezing time in the context trial between GBX-treated 5XFAD mice and saline-treated 5XFAD mice (Fig. 5B, 41.64 ± 12.89% vs. 21.67 ± 8.31%). However, with a comparable exploration time to the saline-treated 5XFAD mice (Fig. 5C), the GBX-treated 5XFAD mice displayed better recognition of familiar objects (Fig. 5D, *p* = 0.0321; raw data in Supplementary Table 2). Unexpectedly, the GBX treatment had no effect on the displaced vs. stationary index, which carries contextual information (Fig. 5E), implying that the intervention time point should be set earlier.

## Discussion

Neuronal hyperactivity is considered an early warning sign of AD [6, 8, 17, 31]. Aβ-related excitotoxicity [32] and calcium overload [18, 33] are reportedly involved in this aberrant neuronal activity. Here, we confirmed that CA1 pyramidal neuronal hyperactivity preceded local extracellular Aβ deposition in the 5XFAD mouse hippocampus, with early AD-like behavioral deficits. Furthermore, the decline in the inhibitory synaptic transmission, rather than the excitatory synaptic transmission and intrinsic properties of the CA1 pyramidal neurons, contributed to aberrant neuronal activation in the early stages of the AD model. The decrease in GABA_A_ receptor localization in hippocampal synapses and the related diminished synaptic response mainly contributed to Aβ-associated hyperactivity (Supplementary Figure 6). Most importantly, memory impairment in the 5XFAD mouse model was partially reversed by GABA_A_ strengthening in vivo. Thus, we propose a novel opinion that functional decline in inhibitory synaptic transmission due to reduced postsynaptic localization of GABA_A_ receptors plays a causative role in hippocampal hyperactivity and ensuing cognitive deficits in the AD process.

We previously found that hippocampus-dependent cognitive ability in the 5XFAD mouse model declined at 4 months of age with the commonly used protocol of the Morris water maze [34, 35]. However, the ability of reversal learning and memory, as well as LTP expression in the hippocampus, declined in the 5XFAD mice compared with their WT littermates as early as 2–3 months old [20], when little extracellular Aβ deposition had developed [34] (Fig. 1F), indicating that synaptic dysfunction associated with early cognitive impairment was a forerunner in the AD process.

Accumulating evidence shows that the dendritic structure is altered in the hippocampal neurons of patients with AD and in animal models [12, 36, 37]. Neuronal hyperexcitability in the 5XFAD mouse hippocampus has been attributed to dendritic structural degeneration [22]. Although CA1 neuronal hyperactivity was confirmed in our study (Fig. 1), we did not find obvious changes in dendritic morphology (Fig. 2), suggesting that functional events rather than structural alterations contribute to the early stages of AD. In consideration of our discovery of unaltered intrinsic excitability of CA1 pyramidal neurons (Fig. 1), we propose that synaptic dysfunction is the real culprit in neuronal hyperactivity occurring during the early stage of AD.

Although we cannot exclude the possibility that CA1 neuronal hyperactivity was initiated by suppression of glutamate reuptake [32], we did not find obvious changes in activity-dependent excitatory synaptic transmission to CA1 pyramidal neurons (Fig. 2). Clinical evidence has shown that tissues isolated from the temporal cortices in the brain of patients with AD display a loss of GABA receptor subunits, α1 and γ2 [38]. In agreement with that study, a decrease in amplitude of IPSCs was observed in hippocampal CA1 pyramidal neurons of the 5XFAD model (Fig. 3), with a small increase in amplitude and frequency of mEPSCs but without pronounced changes in AMPAR EPSCs (Fig. 2). This finding strengthens our proposal that inhibitory inputs to CA1 neurons could be an early dysfunctional event in the AD pathological context. Interestingly, the PPRs revealed an activity-dependent reduction in the inhibitory presynaptic release probability (Fig. 3H), contradicting the increased mIPSC frequency (Fig. 3C), which suggested an enhancement in the spontaneous presynaptic release. The data support an opinion of compensatory remodeling of inhibitory hippocampal circuits, as proposed by Palop et al., although those authors observed an increase in the amplitude of mIPSCs in the dentate gyrus of another AD mouse model [7].

Presynaptic GABA_B_ receptor 1a can bind to sAPPα, which is generated by α-secretase proteolysis of APP, to regulate presynaptic transmitter release [39], or it can interact with APP to stabilize it at the cell surface and thereby reduce Aβ production [40]. Therefore, the decreased activity-dependent presynaptic release probability may be attributed to AD-related mechanisms, e.g., amyloidogenic APP processing, although the mechanism needs further study. Intriguingly, the GABA_A_ α1 distributed in the SLM of the hippocampus was selectively reduced (Fig. 3), with suppressed synaptic fractions and unchanged total protein levels in all hippocampal tissues (Fig. 4). Given that the SLM receives projections from the entorhinal cortex [25], which is the key area involved in early AD pathology [1], we suggest that the aberrant activity of CA1 pyramidal neurons in the early stage of AD may be partially attributed to dysfunction of the entorhinal cortex–CA1 circuit. Nevertheless, how the trisynaptic circuit entorhinal cortex–CA3–CA1 integrates with the direct circuit entorhinal cortex–CA1 should be carefully investigated in future work.

Considering the presynaptic mechanisms, inhibitory interneurons in CA1 local circuits may contribute to network dysfunction under AD conditions, as dysfunction of parvalbumin-positive inhibitory interneurons [3, 41] and oriens-lacunosum-moleculare (O-LM) inhibitory interneuron [42] has been found to be involved in memory impairment in amyloidosis AD models. Importantly, Schmid et al. [42] revealed the axon loss of O-LM interneurons in the CA1 SLM due to a reduction of acetylcholine from septo-hippocampal projections, which supports our finding of decreased PPR of IPSC in CA1 pyramidal neurons (Fig. 3H). Nevertheless, how inhibitory interneurons interact with pyramidal neurons to regulate CA1 local circuits requires further study using a double-patch recording system. Another interesting study demonstrated that Aβ treatment can specifically reduce inhibitory transmission at the dendritic synapses [43], which supports our hypothesis that Aβ-induced abnormal integration of inhibitory synapses in CA1 pyramidal neurons plays a leading role in the AD process.

Although loss of GABA_A_ receptors and inhibitory synaptic dysfunction associated with AD has been previously discussed [38, 44–46], there is no uniform understanding of whether inhibitory synaptic transmission to CA1 pyramidal neurons is altered and involved in the early stage of AD. The α1 subunit-containing GABA_A_ receptors are mainly located on the postsynaptic membrane and mediate phasic inhibition in the brain [29]. Our finding of a loss of function of GABA_A_ receptors in the 5XFAD hippocampus was consistent with clinical evidence that AD patient-derived cell membranes displayed a reduction in GABA-mediated currents [38]. In addition, we found decreased localization of the GABA_A_ α1 subunit in the synaptic membrane but with a normal reserve pool in the hippocampus of 5XFAD mice. Hence, an early therapeutic window during which neuronal activity would be remodeled is proposed. As expected, the GABA_A_ agonist GBX eventually suppressed the neuronal hyperactivity (Fig. 4) and partially prevented cognitive decline (Fig. 5) in the 5XFAD mouse, corroborating that functional impairment in the GABAergic system makes a major contribution to a cognitive abnormality in AD and can be a target for early treatment.

GBX is a hypnotic drug that has been reported to act on extrasynaptic GABA_A_ receptors to drive tonic inhibition in the neocortex [30], thalamus, and dentate gyrus [47]. Here, we suggest that inhibitory synaptic transmission is strengthened by GBX, although we cannot rule out the possible contribution of extrasynaptic GABA_A_ receptor-mediated conductance in this process. Nevertheless, we found no changes in the tonic inhibitory current of CA1 neurons nor in reactive astrocytes (Supplementary Fig. 3), which is reportedly related to local Aβ accumulation in the 5XFAD brain [26, 27]. Notably, a recent study reported that systemic administration of GBX rescued the tau-induced adult hippocampal neurogenesis deficits and improved contextual cognition in AD mice [46]. Together, this evidence suggests that strengthening the GABAergic system would be effective in both the Aβ-related and tau-related courses of AD.

### Limitations

Here, we did not apply systemic treatment in the AD mouse model because we sought to first confirm the direct effect of strengthening GABA_A_ receptors on the abnormal hippocampal network in vivo. Therefore, further studies should be performed to explore a method of AD treatment targeting the GABAergic system that is safe, less toxic, and highly effective.

## Conclusions

In recent years, the fact that many preclinical studies focusing on Aβ have been ineffective has pushed researchers in this field to seek more effective and promising strategies to cure or even prevent AD. Our study suggests that GABAergic remodeling may be a useful path to re-orchestration of the hippocampal network in the early stage of AD, although the following open questions remain to be answered. How does GABA_A_ agonist regulate postsynaptic receptor redistribution? How is the possible interaction of intracellular Aβ and GABA_A_ receptors involved in the AD process? Can activation of GABA_A_ receptors restore functions of the cognitive network in the early stage of human AD?

## Supplementary Information

**Additional file 1: Supporting Information**.

**Additional file 2: Supplementary Table S1**.

**Additional file 3: Supplementary Table S2**.

## Data Availability

The datasets used and/or analyzed during this study are available from the corresponding author on reasonable request.

## Abbreviations

Aβ: β-Amyloid
AD: Alzheimer’s disease
AMPAR: α-Amino-3-hydroxy-5-methylisoxazole-4-propionic acid receptor
AP: Action potential
APP: Amyloid precursor protein
BACE1: β-Site amyloid precursor protein cleaving enzyme 1
EPSC: Excitatory postsynaptic current
FAD: Familial Alzheimer’s disease
GABA: γ-Aminobutyric acid
GABA_A_: γ-Aminobutyric acid A
GBX: Gaboxadol
IPSC: Inhibitory postsynaptic current
mIPSC: Miniature inhibitory postsynaptic current
mEPSC: Miniature excitatory postsynaptic current
NMDAR: *N*-methyl-*D*-aspartate receptor
PPR: Paired-pulse ratio
PV: Parvalbumin
sAP: Spontaneous action potential
SDS: Sodium dodecyl sulfate
SLM: S. lacunosum-moleculare
TTX: Tetrodotoxin
