# Supplementary material for: Reversible GABAergic dysfunction involved in hippocampal hyperactivity predicts early-stage Alzheimer disease in a mouse model
Source: Alzheimers Res Ther. 2021 Jun 14;13:114. doi: 10.1186/s13195-021-00859-8 (PMC8204558; doi:10.1186/s13195-021-00859-8)

**Supporting Information**

**Materials and methods**

**Animal model and brain slices preparation**

APP/PS1 (5XFAD) double transgenic mice (006554, Jackson Laboratory) carry human APP and PS1 transgenes containing five FAD mutations (APPSwFlLon, PSEN1*M146L*L286V) under the transcriptional control of the neuron-specific mouse Thy-1 promoter. The animals were bred in strict accordance with Chinese regulations involving animal protection and were approved by the animal ethics committee of Capital Medical University. Mice were maintained by crossing heterozygous transgenic mice with C57BL/6J wild-type breeders. We used female heterozygous mice in electrophysiological recordings and male heterozygous in behavioral tests, aged between 2.5-3.5 months, and non-transgenic wild-type littermates served as the control group.

Under chloral hydrate (i.p. 300 mg/kg) anesthesia, mice were transcardially perfused with cutting solution, and the brains were immediately removed and cut with vibratome (Leica, VT1200S) in ice-cold, high-sucrose cutting solution containing (in mM): 3 KCl, 1 NaH_2_PO_4_, 26 NaHCO_3_, 0.5 CaCl_2_, 5 MgCl_2_, 213 Sucrose, 10 D-glucose (pH7.4, approximately 300mOsm, bubbled with a mixture of 95% O_2_ and 5% CO_2_). Brains were rapidly removed and attached onto a plate of Leica vibratome, and 300 µm sections were cut in the cold cutting solution. These sections were carefully and rapidly transferred to an artificial cerebrospinal fluid (ACSF) solution at 35^o^C containing (in mM): 130 NaCl, 3 KCl, 1.25 NaH_2_PO_4_, 26 NaHCO_3_, 1 CaCl_2_, 5 MgCl_2_ and 10 glucose (pH7.4, bubbled with 95% O_2_/5% CO_2_) for 40 min. Then the slices were bubbled at room temperature (25^o^C) for a further 20 min, and then moved to a clamp chamber for recording. The CA1 pyramidal neurons were identified on an upright microscope (FN-S2N, Nikon) by structural morphology with obvious dendrites in CA1 strata radiatus.

**Acute hippocampal slice recordings**

For cell recordings, the slices were placed in a recording chamber constantly perfused with ACSF (in mM): 125 NaCl, 5 KCl, 1.2 NaH_2_PO_4_, 26 NaHCO_3_, 2.6 CaCl_2_, 1.3 MgCl_2_,10 glucose, and bubbled continuously with 95% O_2_ and 5% CO_2_. The borosilicate glass was pulled to get patch pipette with resistance of 3-5 MΩ by using a two-step vertical pipette puller PC-10 (Narishige, Japan). We compensated pipette capacitance after formation of high-resistance seal (> 2 GΩ). The R_s_ was monitored throughout the recording procedure by applying a hyperpolarization pulse (-10 mV) either at the initial segment or at the end of a recording course. Any trace with a change in R_s_ by more than 20% was excluded from data analysis. Membrane capacitance (Cm) and R_s_ in each cell were calculated from above -10 mV pulse protocol, with the formula:

Cm = τ•(R_m_+R_s_)/R_m_•R_s_, R_m_ = V_0_/I_m_, R_s_ = V_0_/I_i_,

In this case, V_0_ is 10 mV; I_i_ is the transient current mainly flowing through R_s_, and I_m_ is the steady-state current derived from membrane resistance. Any cells with R_s_ exceeded 30 MΩ were excluded.

Spontaneous action potential (AP) and neuronal intrinsic properties were recorded in current-clamp mode with pipette solution (in mM): 100 K-gluconate, 20 KCl, 10 HEPES, 4 Mg-ATP, 0.5 Na_2_-GTP and 10 Na_2_-phosphocreatine. For intrinsic properties analysis, the following protocols were applied: (1) I-V relationships were obtained from a series of 500 ms current injections ranging from -30 to 160 pA. The input resistance was calculated as the linear slope of the plateau voltage versus current plot from -30 to 30 pA which did not burst AP; (2) short pulse rheobase was measured by a brief 3 ms current injection in steps of 10 pA until the threshold was reached. The resting membrane potential was measured immediately after break-in and current-clamp was acquired, and only cells exhibiting a resting potential between -55 and -75 mV at break-in were used. All neurons with resting potential changes maintained between ±3 mV for the duration of the recordings. For drug treatment, the internal solution (in mM): 143 KCl, 8 NaCl, 1 MgCl_2_, 10 HEPES, 0.4 Na_2_-GTP, 2 Mg-ATP, was used to identify the obvious change in sAP frequency. After 2 min stable baseline recording, the slice was perfused with ACSF containing Gaboxadol (GBX, Sigma, T101) in the final concentration of 5 μM at a speed of 2 ml/min for 10 min, following washing with normal ACSF.

For voltage-clamp configuration, the membrane potential was held at -60 mV, and glass pipettes were filled with (in mM): 130 Cs-methanesulphonate, 10 CsCl, 4 NaCl, 1 MgCl_2_, 10 HEPES, 5 EGTA, 5 Mg-ATP, 0.5 Na_2_-GTP, 12 phosphocreatine (pH7.2-7.3, approximately 265-270 mOsm) for excitatory postsynaptic current (EPSC) recording, whereas with internal solution (in mM): 100 CsCl, 30 N-methyl-D-glucamine, 10 HEPES, 4 NaCl, 1 MgCl_2_, 5 EGTA, 5 Mg-ATP, 0.5 Na_2_-GTP, 12 phosphocreatine (pH7.2-7.3, approximately 265-270 mOsm) for inhibitory postsynaptic current (IPSC) recording. The 0.5 μM tetrodotoxin (TTX) was included in perfusion solution during miniature EPSC (mEPSC) or miniature IPSC (mIPSC) recording. Tonic GABA current was recorded with IPSC pipette solution in the presence of 0.8 µM GABA, 5 µM bicuculline, and 10 µM GABA uptake inhibitor, tiagabine hydrochloride (TGH) in ACSF. The tonic current was determined by the change of holding current after perfusion of ACSF containing above drugs. In evoked current recording, 2 mM QX-314 was added in pipette solution. Evoked IPSCs (eIPSCs) were recorded at -60mV in the presence of 20 µM CNQX and 50 µM AP-5. Evoked EPSCs (AMPAR-EPSCs) were recorded in the presence of 50 µM AP-5 and 100 µM picrotoxin. Concentric bipolar electrode (CBBEB75, Frederick Haer) triggered with an impulse isolator (A-M system 2100) was placed 100 µm away from the recording neuron in the CA1 strata radiatus in the same plane. To measure the input-output curves for pyramidal neurons, a sequential increased stimulus strength from 10 µA to 100 µA was applied. Each stimulus was repeated 3 times at stimulus interval of 15 s, and the average response under the stimulus strength was subjected to statistical analysis. Paired-pulse ratio was calculated as a ratio of the second current peak under pulse2 (P2) to the first (P1), separated by interstimulus intervals of 20, 50, 100 and 200 ms, respectively. The stimulus strength for PPR was less than half of the maximum output in I-O protocol.

All recordings were using HEKA EPC10 and data sampling was set at 10 KHz for voltage-clamp, 20 KHz for current-clamp, respectively. Data analysis was accomplished by pClamp10.4 (Axon instruments) for current-clamp signal and evoked current, or Mini analysis software (Version 6.0.7) for mEPSC and mIPSC recordings. Miniature events were determined first with the software by template matching search, and a minimal threshold was set at 5 pA, and then each event was visually chosen for inclusion or exclusion by an experimenter blind to the sample sets.

**Subcellular fractionation**

Shared with the above electrophysiological experiment when needed. Fractions were prepared as described previously [1] with a few modifications. Briefly, the right cortex and hippocampus were rapidly isolated and homogenized in cold lysis buffer (10 ml/g, 15 mM Tris (pH 7.6), 0.25 M sucrose, 2 mM EDTA, 1 mM EGTA, 10 mM Na_3_VO_4_, 25 mM NaF, 10 mM sodium pyrophosphate), containing 1 mM phenylmethylsulfonyl fluoride (PMSF), protease and phosphatase inhibitors (78443, Thermo Scientific). After centrifugation at 800 g for 5 min at 4^o^C, 100 μL of supernatant was collected as total protein (T). The remaining supernatant was subjected to 10,000 g for 10 min at 4^o^C, and supernatant was collected to get a soluble cytosolic fraction (S) by the next ultracentrifugation at 165,000 g for 30 min at 4^o^C, while the pellet was lysed by 1% Triton buffer (lysis buffer above containing 1% TritonX-100, 300 mM NaCl), followed by centrifugation at 16,000 g for 30 min at 4^o^C to get supernatant as the cytosolic protein in synapses (P1), and pellet dissolved in 1% SDS as the membrane-associated protein in synapses (P2). These fractions were loaded with SDS loading buffer (5X, P0015, Beyotime) and subjected to western blotting.

**Western blotting**

Equal amounts of proteins, which were quantified with BCA protein assay kit (23225, Pierce TM) from WT and FAD fractions were subjected to 10% SDS-polyacrylamide gels and transferred to nitrocellulose membranes. The membranes were blocked with 5% nonfat dry milk dissolved in PBST buffer (8 mM Na_2_HPO4, 0.136 M NaCl, 2 mM KH_2_PO_4_, 2.6 mM KCl, 0.05%(V/V) Tween-20) for 1 h at RT. Then the membranes were incubated with various primary antibodies (α1 subunit, 1:1000, Abcam, ab33299; γ2 subunit, 1:1000, SYSY, 224003; GluA1, 1:1000, Sigma-Aldrich, AB1504; GluA2, 1:1000, MAB397, Merck Millipore) overnight at 4^o^C. After washing three times, membranes were incubated with secondary antibodies: HRP conjugated goat anti-mouse IgG (1:10000, CW0102A, CWBIO) or anti-rabbit IgG (1:10000, CW0103A, CWBIO), followed by enhanced chemiluminescence substrate (Thermo Scientific, 32106) incubation to develop positive bands. Blots quantification was determined by densitometric measurements with Image J software.

**ELISA**

After decapitation, the mouse brain was removed and quickly frozen in liquid nitrogen. Tissues were homogenized in cold RIPA buffer (50 mM Tris (pH 7.4), 150 mM NaCl, 1% TritionX-100, 1% sodium deoxycholate, 0.1% SDS) containing protease and phosphatase Inhibitors (1:100, 78443, Thermo Scientific) for 30 min on ice, followed by centrifugation at 13,000 rpm for 15 min at 4^o^C. The supernatant was prepared for soluble Aβ detection, and the pellet was dissolved in lysis buffer containing 8% SDS, 8 M urea and 5 mM EDTA to measure insoluble Aβ level. The soluble and insoluble fractions were quantified by human Aβ42 and Aβ40 kits (KHB3441, KHB3481, respectively, Invitrogen), according to the manufacturer’s protocol. All values were normalized to the total protein level determined by BCA assay.

**Biocytin labeling and immunostaining**

Neuronal morphology and spine density of CA1 neurons were determined by adding biocytin (0.4%) in the pipette solution during whole-cell recording. After recordings were finished, slices were fixed overnight in paraformaldehyde (4%), permeated by 0.5% Triton X-100 for 20 min, and stained using streptavidin-coupled Alexa 488 (Invitrogen) for 2 hr at RT. To quantify spine density, 3D morphological analyses of neurons were obtained from image stacks of 100 µm at Z step of 0.5 µm imaged by a 60 X oil objective lens (Olympus, FV3000) and reconstructed using Imaris filament tracer (Bitplane). During morphometric analyses, the experimenter was blinded to the mouse genotype.

For immunofluorescent detection of GABA_A_ receptors and AMPA receptors, the mouse brain was removed after anesthesia and transcardiac perfusion with saline and paraformaldehyde (4%) sequentially. The 30 µm thick brain sections were cut with vibratome (Leica VT1000S) in phosphate buffered saline (PBS). After permeated with 0.3% Triton X-100 for 30 min and blocked with 10% goat serum for 30 min, slices were incubated with primary antibodies (α1 subunit, 1:200, Abcam, ab33299; γ2 subunit, 1:200, SYSY, 224003; GluA1 subunit, 1:200, Sigma-Aldrich, AB1504; MAP2: mouse anti-MAP2 antibody, 1:500, MAB3418, Sigma or Rabbit anti-MAP2 antibody, 1:500, ab32454, Abcam) at 4^o^C overnight, followed by incubation with Alexa Fluor 488 (1:500, Invitrogen, A11008 or A11001), Alexa Fluor 594 (1:500, Invitrogen, A11037 or A11032). Slices were covered with microscope cover glasses. Two sections from the WT brain and FAD brain, respectively, were attached to a slide, and three pairs of WT/FAD brains were used for confocal imaging of GluA1 or GABA_A_ α1 antibody recognition. After staining, all sections were imaged under the same criterion with Confocal Laser Scanning Microscope (Olympus FV3000). The optimal focal plane was divided into 18 layers with Z-stack, and 1 µm step scanning was set under 60X oil objective lens. Then processed images were subjected to 8-bit analysis with ImageJ, in which background subtraction and thresholding were performed. Mean gray value (integrated density divided by area of ROI (regions of interest)) was calculated and subjected to statistical analysis.

Avidin-Biotin complex (ABC) staining (Vector, PK-4002) was used to determine Aβ positive signals. What was different from above mentioned is: blocked by 3% hydrogen peroxide for 30 min; 10% horse serum incubation; primary anti-β-Amyloid 1-16 antibody (6E10, 1:1000 diluted in 10% horse serum, Covance, SIG-39300) at 4^o^C incubation; biotinylated horse anti-mouse IgG for 1 h, followed by a mixture of avidin DH (Reagent A) and its paired biotinylated enzyme (Reagent B) for another 1 h incubation; the positive signals were detected by diaminobenzidine (DAB) chromogenic substrate reaction; slices were air-dried and dehydrated in ethanol with 60%, 70%, 80%, 90%, 100% concentration sequentially, and hyalinized with xylene; slices were imaged by Olympus VS120 virtual microscopy slide scanning system. The plaque intensity level per unit area was analyzed with Image J software.

**Electron Microscopic Imaging**

Anesthetized mice were perfused with saline and 4% PFA in PBS buffer containing 0.075% glutaraldehyde. The brains were quickly removed and coronal sections (1 mm) were cut by using a pre-cooled mouse brain matrice, then the interested brain fields were extracted out by a punch (in 1 mm diameter). These punched tissues were immersed in 2.5% glutaraldehyde for 2 h post-fixation at 4^o^C, and followed by washing three times with 0.1 M PB to stop fixation. The 2.5% glutaraldehyde was prepared by mixing 9 ml 0.1 M phosphate buffer (PB) and 1 ml glutaraldehyde of 25%. The samples were embedded in resin and cut on a microtome (Leica EM, UC7) and post-stained with uranyl acetate and lead citrate. The ultra-thin sections were observed by a transmission electron microscope (HITACHI, JAPAN, HT7700). The asymmetric synapse was identified by a distinct postsynaptic density, presynaptic vesicles and an obvious synaptic cleft. Total synapses in images at 8,000 x primary magnification were subjected to parameter measurements by an observer blinded to the genotype of the sample.

**Behavior and drug treatment**

Contextual fear conditioning (CFC)

All mice were habituated to the testing room for 1 hour. As previously described [2], briefly, on day 1, the mouse was placed in the chamber A (a chamber with a grid floor, opaque triangular ceiling and scented with 5% acetic acid) for 3 min to record the baseline activity, then treated by a conditioned stimulus (CS), which consisted of continuous tone (4000 Hz, 80 dB, 30 s). During the last 2 s of CS period, the mouse was exposed to an unconditioned stimulus (US), i.e. a continuous foot shock (0.6 mA). This CS-US protocol was repeated 3 times with a 60 s inter-trial interval. Following the last shock, the mouse was left in the chamber for another 1 min before return it to the home cage. On day 2, contextual conditioning test was performed by placing the mouse in chamber A for 3 min. Two hours later, the mouse was put in the chamber B (a chamber with a smooth board and without a triangular ceiling and acetic acid) for 3 min to record baseline state, and then exposed to the same tone as that in chamber A but without any foot shocks, i.e., cued test. The chambers were cleaned with 10% alcohol after each mouse was trained or tested. The percent of time freezing of the mouse was subjected to statistical analysis.

Episodic-like memory

According to previous description [3], mice were habituated in a test room for 3 days, 30 min/day, and tested in a white acrylic chamber (L*W*H, 20*20*18 cm). Briefly, at day 4, each mouse received two sample trials and one test trail. The chamber was marked at the four walls by different icons. On the first sample trial, the mouse was placed at the center of the chamber with 4 same objects (old object) arranged in a triangle-shaped configuration, and given 10 min to explore. After 50 min, the mouse was placed in chamber with another 4 same objects (recent object) arranged in a quadrated configuration, and allowed to explore for 10 min. After 50 min, the mouse was placed in a test chamber with two old objects and two recent objects (Figure 1C), and allowed 10 min to explore. After each exploration, the chamber was cleaned by 75% alcohol. Exploration time on each object in test trial, i.e., discrimination trial was recorded for analysis. Videos were captured by a digital video, and analyzed by Ethovision 10.0 software (Nodlus).

Intraventricular delivery of GBX

Intraventricular delivery was performed as previously described [4]. The 5XFAD male mice at 3.5-month-old were randomly divided into two groups: normal saline (NS) treatment and GBX treatment (in final local concentration of 5 µM). The unilateral intraventricular drug delivery was applied by osmotic pumps (ALZET, 2004) at a speed of 0.25 µL/h for 28 days. Briefly, fully assembled pumps with catheters were primed in sterile saline for 40 h at 37^o^C before animal treatment. Mice were anesthetized with a mixture of ketamine and xylazine (0.1 mg/g, 0.01mg/g, respectively, i.p.), and put on stereotaxic apparatus. An incision from base neck to middle point between mouse eyes was made, and the pump was inserted into the skin at the neck base and pushed toward the left hindlimb. Using stereotaxic apparatus we determined the right intraventricular point (AP: -0.5 mm; ML: 1.1 mm) and drilled the skull and inserted the cannula into the drilled hole, fixed the cannula by glue on skull, followed by suturing. After 28 days, behavior tests were performed.

References

1. Gu Z, Liu W, Yan Z. {beta}-Amyloid impairs AMPA receptor trafficking and function by reducing Ca2+/calmodulin-dependent protein kinase II synaptic distribution. J Biol Chem 2009;284:10639-10649.

2. Martinelli DC, Chew KS, Rohlmann A, Lum MY, Ressl S, Hattar S, et al. Expression of C1ql3 in Discrete Neuronal Populations Controls Efferent Synapse Numbers and Diverse Behaviors. Neuron 2016;91:1034-1051.

3. Dere E, Huston JP, De Souza Silva MA. Episodic-like memory in mice: simultaneous assessment of object, place and temporal order memory. Brain Res Brain Res Protoc 2005;16:10-19.

4. DeVos SL, Miller, T.M. Direct Intraventricular Delivery of Drugs to the Rodent Central Nervous System. J. Vis. Exp. 2013;DOI 10.3791/50326 (2013)e50326.

**Supplementary Figures**


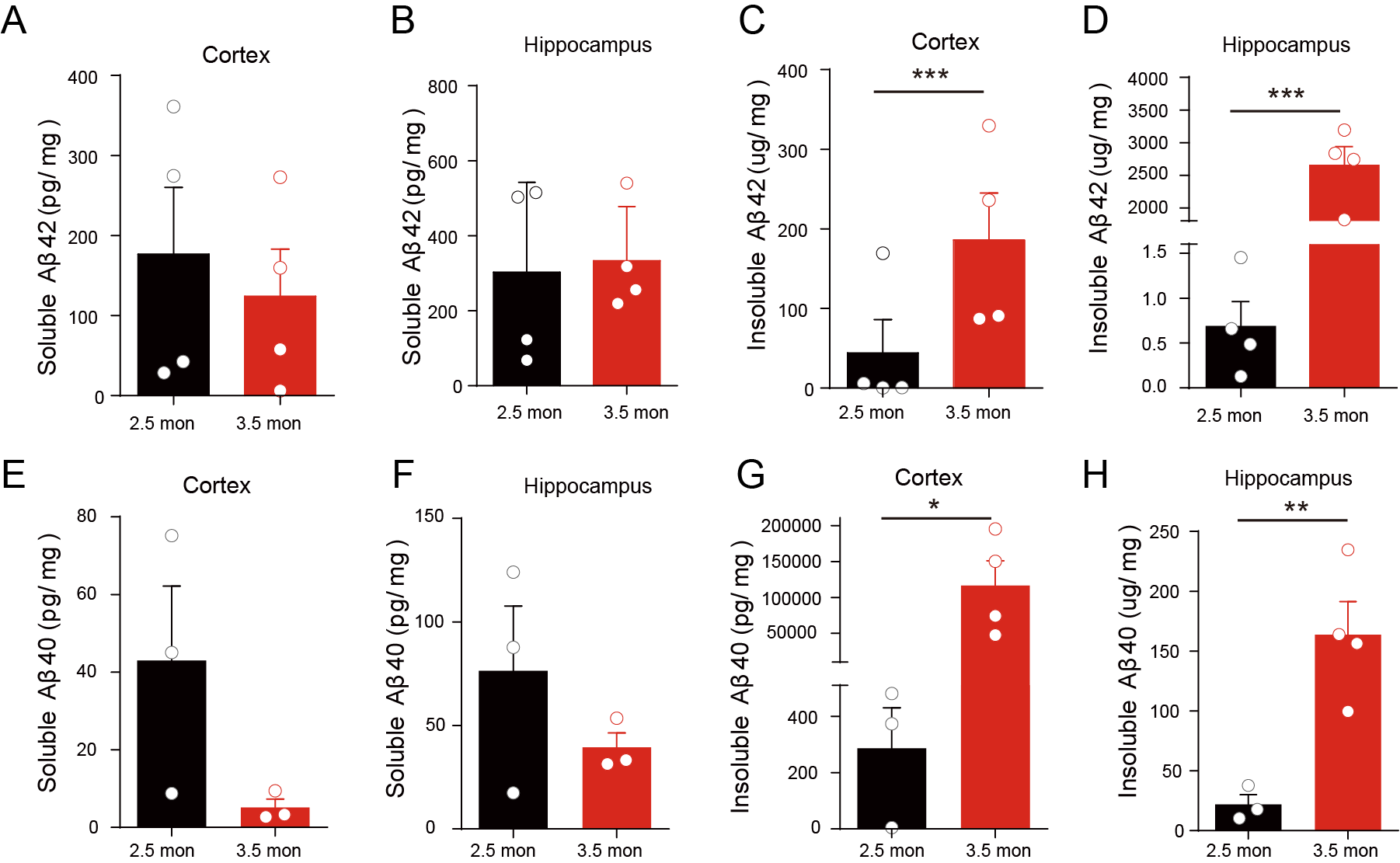


**Supplementary Figure 1. The 5XFAD mice showed a dramatic increase in insoluble Aβ in cortex and hippocampus in age of 2.5-3.5 months old.** **A-D,** soluble Aβ42 in cortex (A) or hippocampus (B) and insoluble Aβ42 in cortex (C) or hippocampus (D) were detected by ELISA kit for human Aβ42 (KHB3441, Invitrogen). **E-H,** soluble Aβ40 in cortex (E) or hippocampus (F) and insoluble Aβ40 in cortex (G) or hippocampus (H) were detected by ELISA kit for human Aβ40 (KHB3481, Invitrogen). All values are presented as mean ± SEM, unpaired student’s t-test, *p < 0.05, **p < 0.01, ***p < 0.001 vs. 2.5 mon. Dots show number of mice.


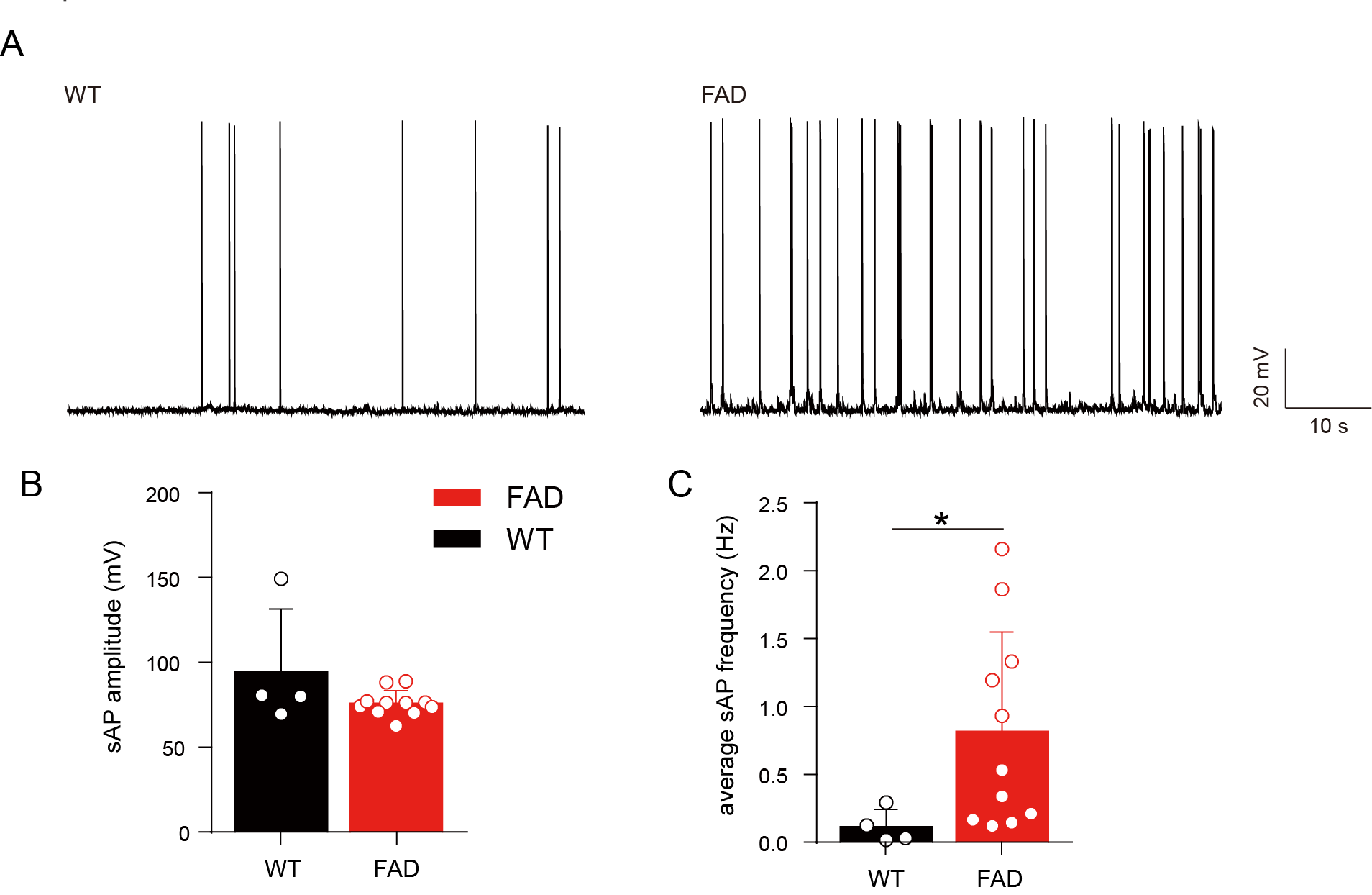


**Supplementary Figure 2. The 5XFAD exhibited obvious CA1 pyramidal neuronal hyperactivity at age of 5 months. A,** representative trace of spontaneous action potential (sAP) of CA1 pyramidal neurons in WT or 5XFAD mouse. Bar scale is 20 mV, 10s. **B,** the sAP amplitude of individual neuron was subjected to statistical analysis. **C,** the sAP frequency per neuron during 10 min recording was averaged and statistically analyzed. All values are presented as mean ± SEM, unpaired student’s t-test, *p < 0.05 vs. WT. Dots show number of neurons.


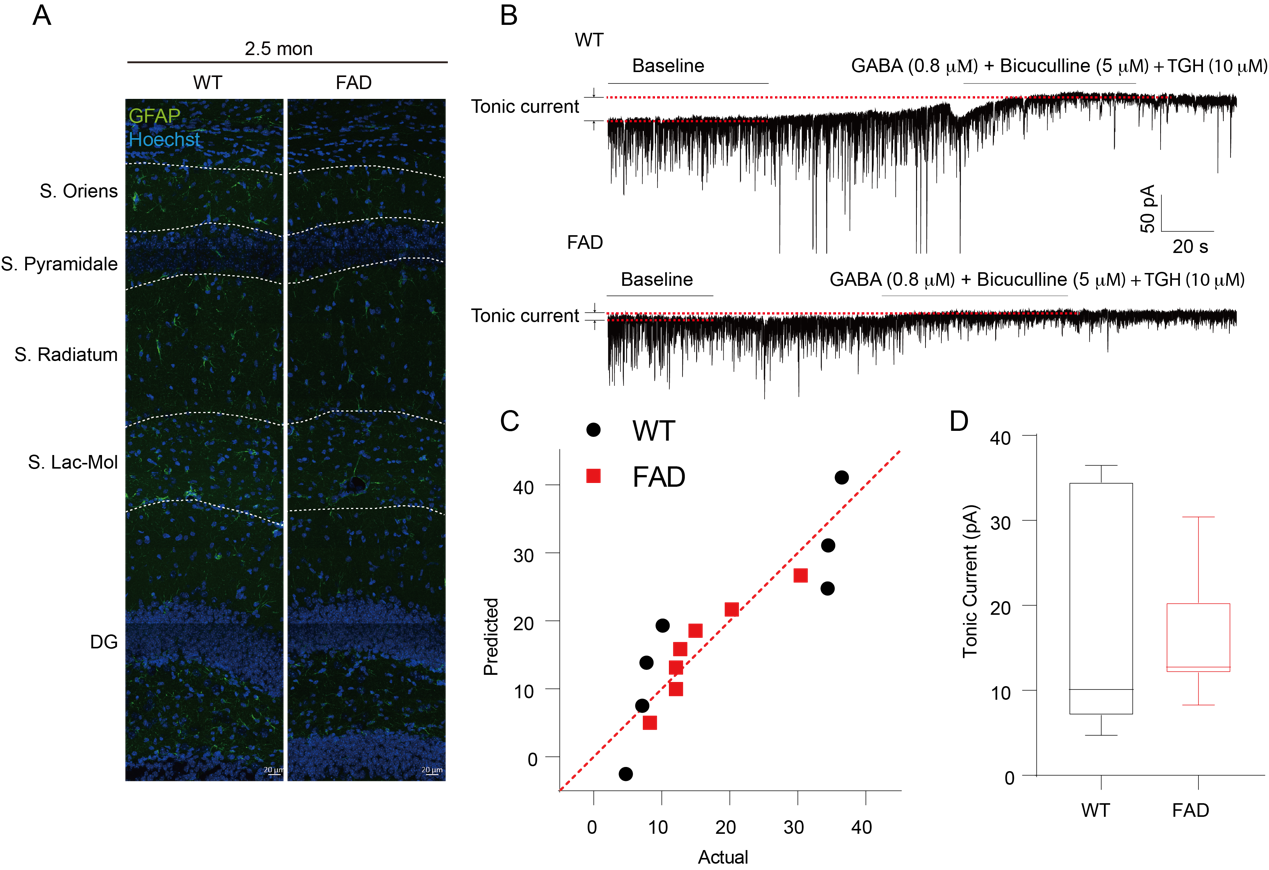


**Supplementary Figure 3. AD transgenic background has no effect on astrocytic reactivity and tonic inhibitory current of CA1 pyramidal neurons in 5XFAD mice in early stages. A,** representative confocal images of glial fibrillary acidic protein (GFAP) staining, which is specific for recognition of astrocytes in WT and 5XFAD brain slices (green). Nucleus were stained by Hoechst (blue). Hippocampal areas were separated by dashed line. S. Lac-Mol stands for S. lacunosum moleculare, and DG stands for dentate gyrus. Bar scale is 20 µm. **B,** representative traces of tonic current from WT or FAD CA1pyramidal neuron. The value of tonic current was determined by the change of holding current from baseline to after perfusion of ACSF containing GABA (0.8 µM), bicuculline (5 µM) and tiagabine hydrochloride (TGH) in final concentration of 10 µM. The membrane potential was held at -60 mV. Bar scale: 50 pA, 20 s. **C,** the data passed Kolmogorov-Smirnov normality test, and the plot shows distribution of individual value of tonic current. **D,** statistical analysis (student’s t-test) shows no significant changes in tonic currents between WT (19.30 ± 14.89 pA) and 5XFAD (15.85 ± 7.40 pA). Data are presented as mean ± SD, n = 7 neurons/7 slices/5 mice.


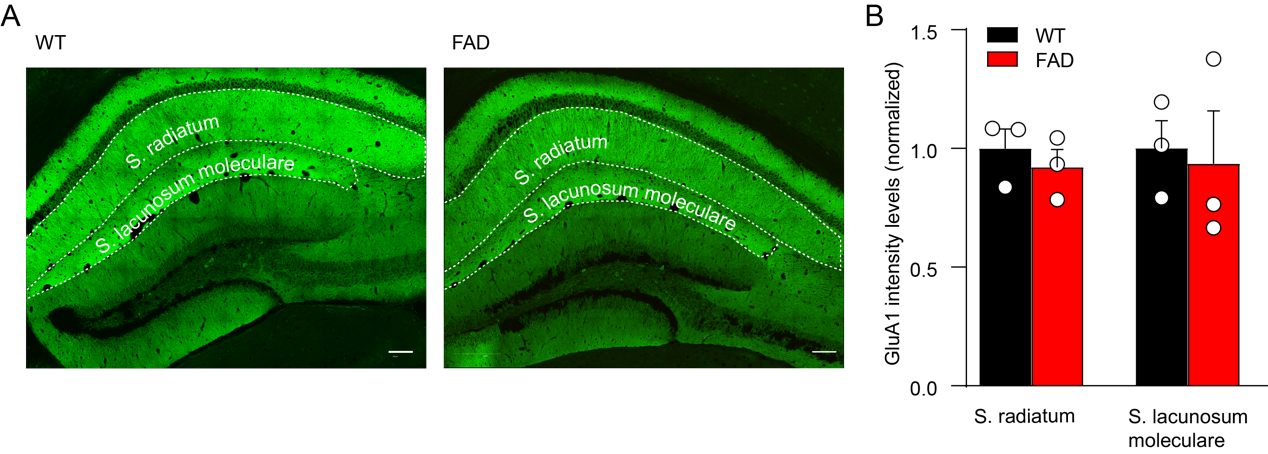


**Supplementary Figure 4. GluA1 expression in hippocampus of 5XFAD mouse at 2.5-month-old was not altered by AD-like contexts. A,** representative images of GluA1 staining by anti-GluA1 antibody (1:200, AB1540, Sigma-Aldrich), followed by Alexa Fluor 488 conjugated second antibody (1:500, A11008, Invitrogen) recognition. The images were captured with confocal microscope (FV3000, Olympus). Areas surrounded by dashed lines show S. radiatum and S. lacunosum moleculare of hippocampus in WT or 5XFAD mouse brain. Bar scale is 100 µm. **B,** mean gray value (integrated density normalized to interested area) was subjected to statistical analysis. Data are presented as mean ± SEM, unpaired student’s t-test, and dots indicate n = 3 mice.


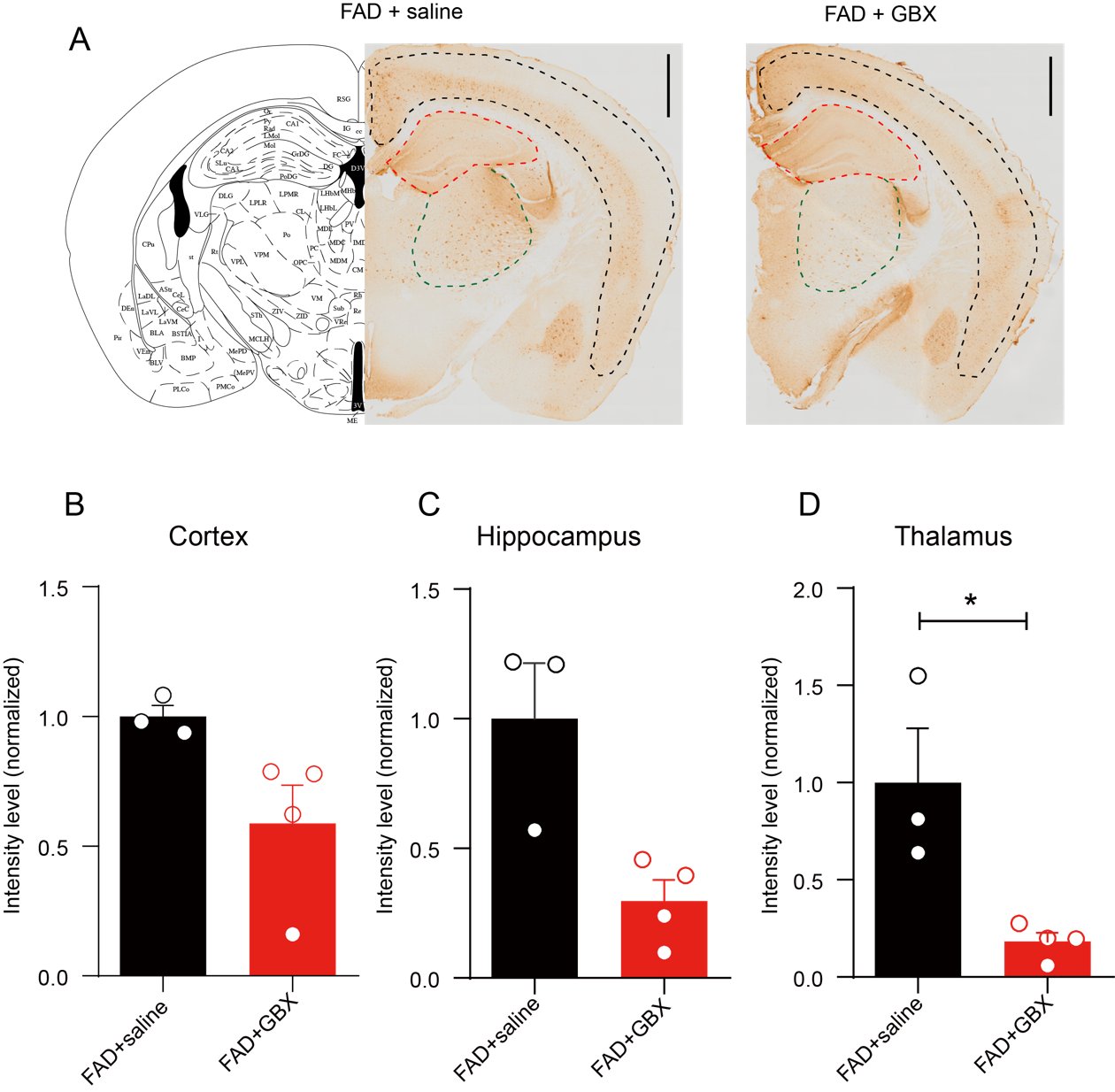


**Supplementary Figure 5. Activating GABA_A_ receptors reduced Aβ deposition in 5XFAD mouse brain.** **A,** the Aβ positive plaques were detected by method of Avidin-Biotin complex (ABC) staining (Vector, PK-4002), and mouse monoclonal anti-6E10 antibody was used to recognize Aβ. The images were captured with Olympus VS120 virtual microscopy slide scanning system (20X object lens). Regions of interested (ROI) were subjected to Aβ-positive plaque intensity level measurement with Image J software. The area surrounded by dashed line indicates a certain region in whole brain, black for cortex, red for hippocampus and dark green for thalamus. Bar scale is 1 mm. The mouse brain atlas (left panel) shows the specific regions of positive signals location. **B-D,** statistical analysis of Aβ-positive plaque intensity levels (normalized) in cortex (B), hippocampus (C) and thalamus (D) of 5XFAD mice treated with saline (NS) or Gaboxadol (GBX) intraventricular delivery at speed of 0.25 µL/h for 28 days. All values are presented as mean ± SEM. Student’s t-test was used, *p < 0.05 vs. FAD+NS. Dots in graph indicate n = 3 mice in FAD+NS, 4 mice in FAD+GBX.


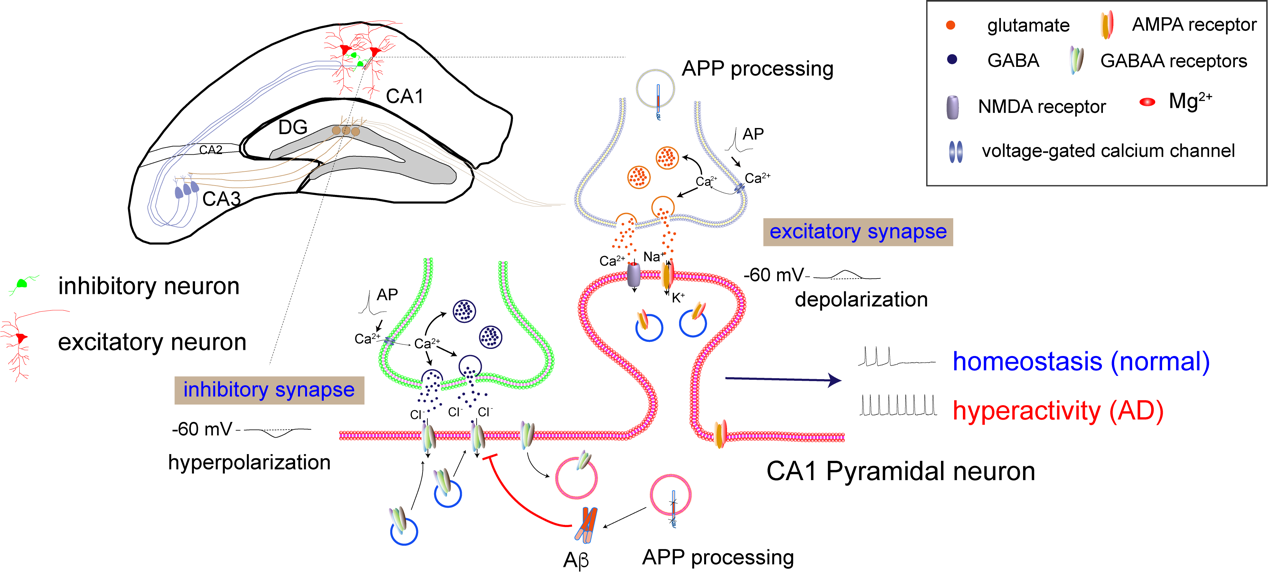


**Supplementary Figure 6. Working model of GABAergic postsynaptic mechanism involved in hippocampal hyperactivity in the early stage of AD.**

The CA1 pyramidal neuron is the major executor for functional circuit of the hippocampus, which is maintained by a balance between inhibitory and excitatory synaptic transmission. In the process of AD pathology, Aβ initially accumulated intracellularly in the CA1 neuron, meanwhile, postsynaptic membrane localization of GABAA receptors is decreased. Thus, the inhibitory synaptic transmission onto the CA1 pyramidal neuron was attenuated, while excitatory inputs keep compensatory. This inhibitory/excitatory transmission imbalance results in hyperactivity of the CA1 pyramidal neuron, ultimately resulting in hippocampal network aberrance.

Full gels for Figure 5B, C , D, E. The GluA2 bands were developed from the same gel as GABAA γ2 bands.


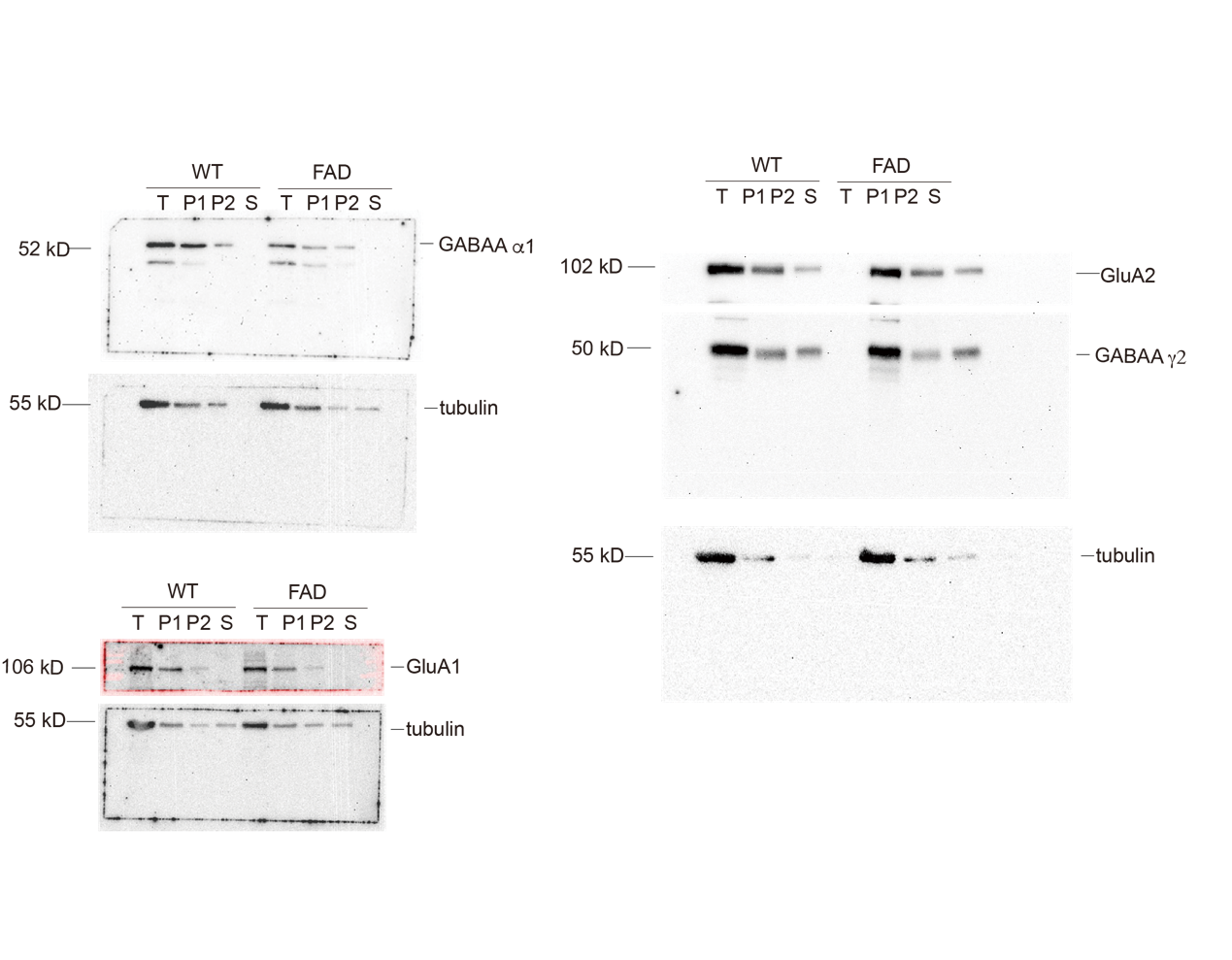

Supplement: Supplementary file 1 — Additional file 1: Supporting Information. [file 13195_2021_859_MOESM1_ESM.docx]
